# Listening to speech with a guinea pig-to-human brain-to-brain interface

**DOI:** 10.1038/s41598-021-90823-1

**Published:** 2021-06-10

**Authors:** Claus-Peter Richter, Petrina La Faire, Xiaodong Tan, Pamela Fiebig, David M. Landsberger, Alan G. Micco

**Affiliations:** 1 Department of Otolaryngology, Northwestern University, 320 E. Superior Street, Searle 12-561, Chicago, IL 60611 USA; 2 Department of Biomedical Engineering, Northwestern University, 2145 Sheridan Road, Tech E310, Evanston, IL 60208 USA; 3 Department of Communication Sciences and Disorders, Northwestern University, Evanston, IL 60208 USA; 4 The Hugh Knowles Center for Clinical and Basic Science in Hearing and Its Disorders at Northwestern University, Evanston, IL 60208 USA; 5 Department of Otolaryngology, New York University Grossman School of Medicine, 550 First Avenue, New York, NY 10016 USA; 6 Department of Otolaryngology, Northwestern University Feinberg School of Medicine, Searle Building 12-470, 303 E. Chicago Avenue, Chicago, IL 60611-3008 USA

**Keywords:** Neurophysiology, Neural decoding, Neural encoding

## Abstract

Nicolelis wrote in his 2003 review on brain-machine interfaces (BMIs) that the design of a successful BMI relies on general physiological principles describing how neuronal signals are encoded. Our study explored whether neural information exchanged between brains of different species is possible, similar to the information exchange between computers. We show for the first time that single words processed by the guinea pig auditory system are intelligible to humans who receive the processed information via a cochlear implant. We recorded the neural response patterns to single-spoken words with multi-channel electrodes from the guinea inferior colliculus. The recordings served as a blueprint for trains of biphasic, charge-balanced electrical pulses, which a cochlear implant delivered to the cochlear implant user’s ear. Study participants completed a four-word forced-choice test and identified the correct word in 34.8% of trials. The participants' recognition, defined by the ability to choose the same word twice, whether right or wrong, was 53.6%. For all sessions, the participants received no training and no feedback. The results show that lexical information can be transmitted from an animal to a human auditory system. In the discussion, we will contemplate how learning from the animals might help developing novel coding strategies.

## Introduction

It is a human dream to communicate directly from brain-to-brain, control machines by direct brain-to-machine connections, or use devices serving as an input to the neural system^1–4^. Especially, brain-to-brain communication is intriguing and has been tried successfully in the past. Previous human-to-human or human-to-animal interfaces used various, non-corresponding brain areas or sensory systems to show information transfer between the brains.

In one experiment, two players participated in a computer game. The game’s task was to defend a city from rocket attacks by shooting down the rockets with a cannon before reaching the city while avoiding the shooting down of friendly flying objects. While one participant (the sender) had visual control over the game but no touchpad to activate the cannon, the second participant (the receiver) had only the touchpad but could not see the game. Verbal or visual communication among the test subjects was impossible because the participants were in different buildings, separated by about one mile. Instead, electroencephalography (EEG) was used to record signals from one human test subject, transmit the information via the internet, and stimulate a second subject's brain through transcranial magnetic stimulation^3^. The brain-to-brain communication was determined by the computer game's performance and showed a rudimentary form of direct information transfer from one human brain to another^3^.

In a different experiment, Yoo and coworkers used the EEG recordings evoked by a visual flicker stimulus to control rats' brains via transcranial focused ultrasound and showed that the flicker response translates into the rat's tail movement^5^.

Matching brain areas were used to study brain-to-brain communications in rats. During the experiment, neural activity was recorded in one rat (coder rat) while performing a task. The activity was then converted into electrical pulses to stimulate a second rat's matching brain areas (decoder rat). The second rat showed similar behavior as the coder rat guided solely by the coder rat’s brain^6^. Note, in this experiment, the sender and the receiver animals were both rats.

The published work has demonstrated that information can be transmitted directly among brains. However, from the previously published experiments, it is not clear whether the sender and receiver brain must be from the same species for successful information transmission. We hypothesize that a rodent brain can serve as a surrogate to process information, and the processed information is intelligible to the brain of a different species, such as humans. This hypothesis was tested in this study in the auditory system of guinea pigs and humans. The animals were the coder, and human cochlear implant (CI) users were the decoder. Neural activity recorded in the brain, the central nucleus of the guinea pig inferior colliculus (ICC), was played back to human test subjects via their CI. The results of the study show that speech information processed by a rodent brain is intelligible for humans. The results of these experiments suggest that neural networks can exchange information among brains and form the base for the development of biological computing devices and the design of more powerful brain-to-machine interfaces. The findings may also impact the development of coding strategies in CIs because neural responses obtained from the animals could serve as a model for pulse patterns delivered by future CI coding strategies.

## Results

### Neural recordings

**Figure 1:**
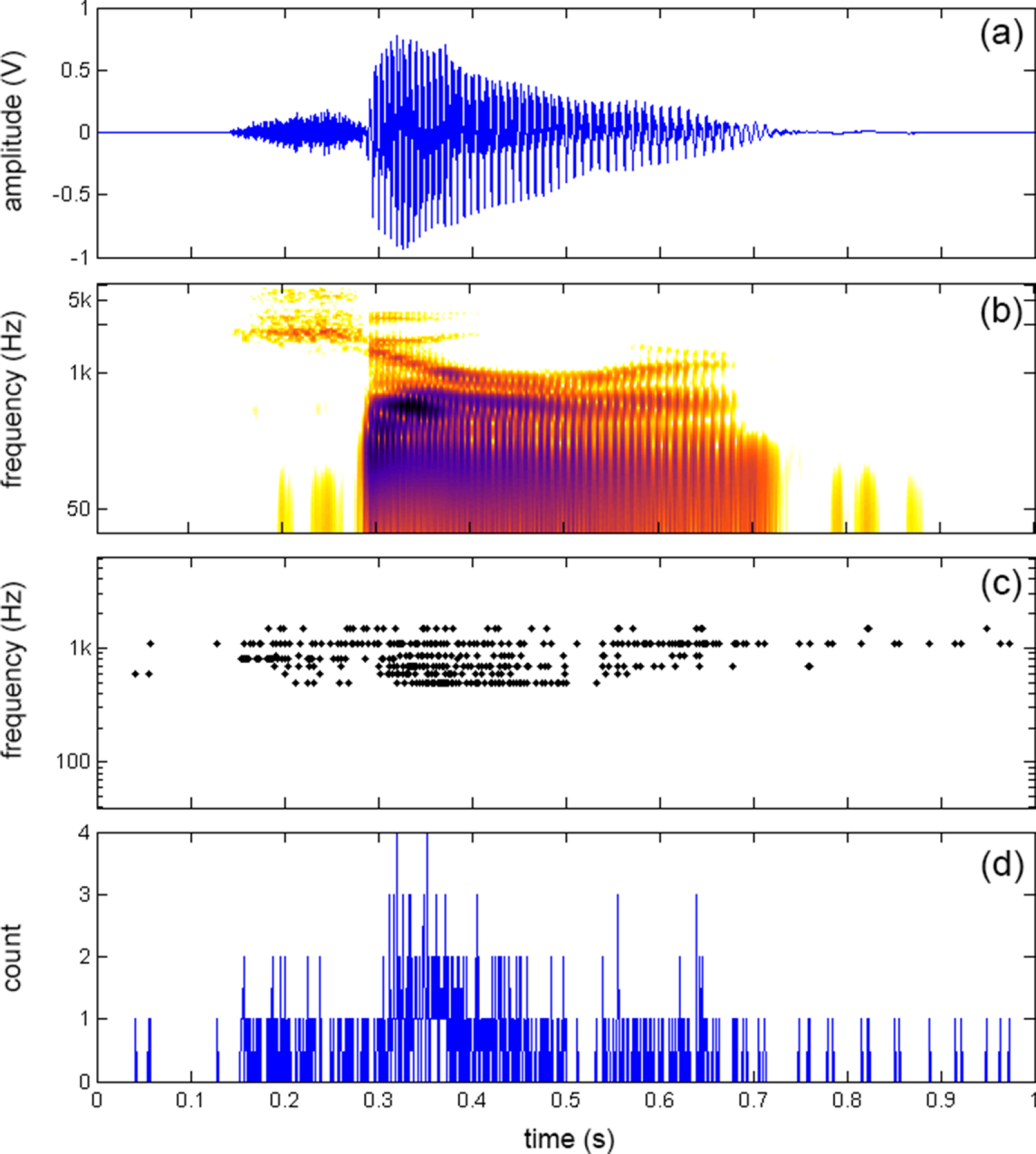
(**a**) Shows the waveform of the word “shore” and (**b**) its corresponding spectrogram. (**c**) Shows the spike raster plot of the recorded neurons played to subjects 5 to 9. The maximum rate was 132 pulses per second (pps), the average rate 58.5 ± 35 pps. The frequency map presented to these subjects was composed of 6 different frequency channels ranging from 500 to 1500 Hz. (**c**) shows the sum of all spikes across the seven channels over time. Note the bin size is ~ 22 µs.

Single units with different characteristic frequencies (CFs) were recorded from the guinea pig’s ICC's central nucleus in response to speech signals. Figure 1 shows the waveform (Fig. 1a) and the corresponding spectrogram (Fig. 1b) of the word “shore”. The spike raster plots' multiple lines represent the neural recordings at six different CFs, mimicking the patterns found in the words' spectrograms (Fig. 1c). Electrical pulse trains generated with a timing pattern based on these spike raster series served as the signals fed into different frequency channels of the patients' CIs. Note, frequency content below 500 Hz is underrepresented due to the challenge of recording from neurons with a low CF in the ICC. The lack of channels available to transmit information limited the data presented to the patients.

The temporal and spectral spike rates can also be investigated by analyzing the spike patterns shown in the spike raster plot. Figure 1d shows for the word “shore” the temporal sum, which is the number of spikes occurring at a selected time summed over all channels. This temporal sum mimics the original wave file's amplitude and shows that concurrent pulses occur within the speech signal. The rate is still low, though, not exceeding 6 with a bin size of 22 µs for any of the words presented throughout all trials. The spectral spike rates, defined as the pulses per second (pps) for one frequency channel, are much lower than that of current CIs. The average spike rate of the information presented to patients was 42.6 ± 31.9 pps with a maximum of 149 pps. The instantaneous rate presented to patients had a maximum of 1760 pps with a mean of 400 ± 412 pps. The upper limit of the rate pitch is not a concern because the stimulus presented via the CIs does not contain a carrier with a fixed stimulation rate. The electrical pulses occur at a stochastic time pattern with a fixed amplitude, which decreased the total charges delivered to a patient compared to the charges delivered using a conventional CI coding strategy. Frequency information is given by the electrode contact location and the sound intensity by the pulse rate. It is unlikely that rate encodes pitch (rate-pitch) as suggested for contemporary coding strategies because the pulse patterns have a Poisson-like distribution with preferred intervals with a difference between maxima equal to 1/CF of the frequency band under investigation. It is unclear whether patients can determine the mode of inter-pulse-time-intervals. The rate reported in this study is the average rate of pulses presented through a CI. Contemporary CIs’ coding strategies differ; they modulate a carrier with a fixed rate with the acoustic signal's envelop information. Differences in spike rate can be attributed to the recorded neurons' activity, though some fluctuation is due to differences in down sampling across testing periods.

### Patient trials

#### Description of the transferred information

**Figure 2:**
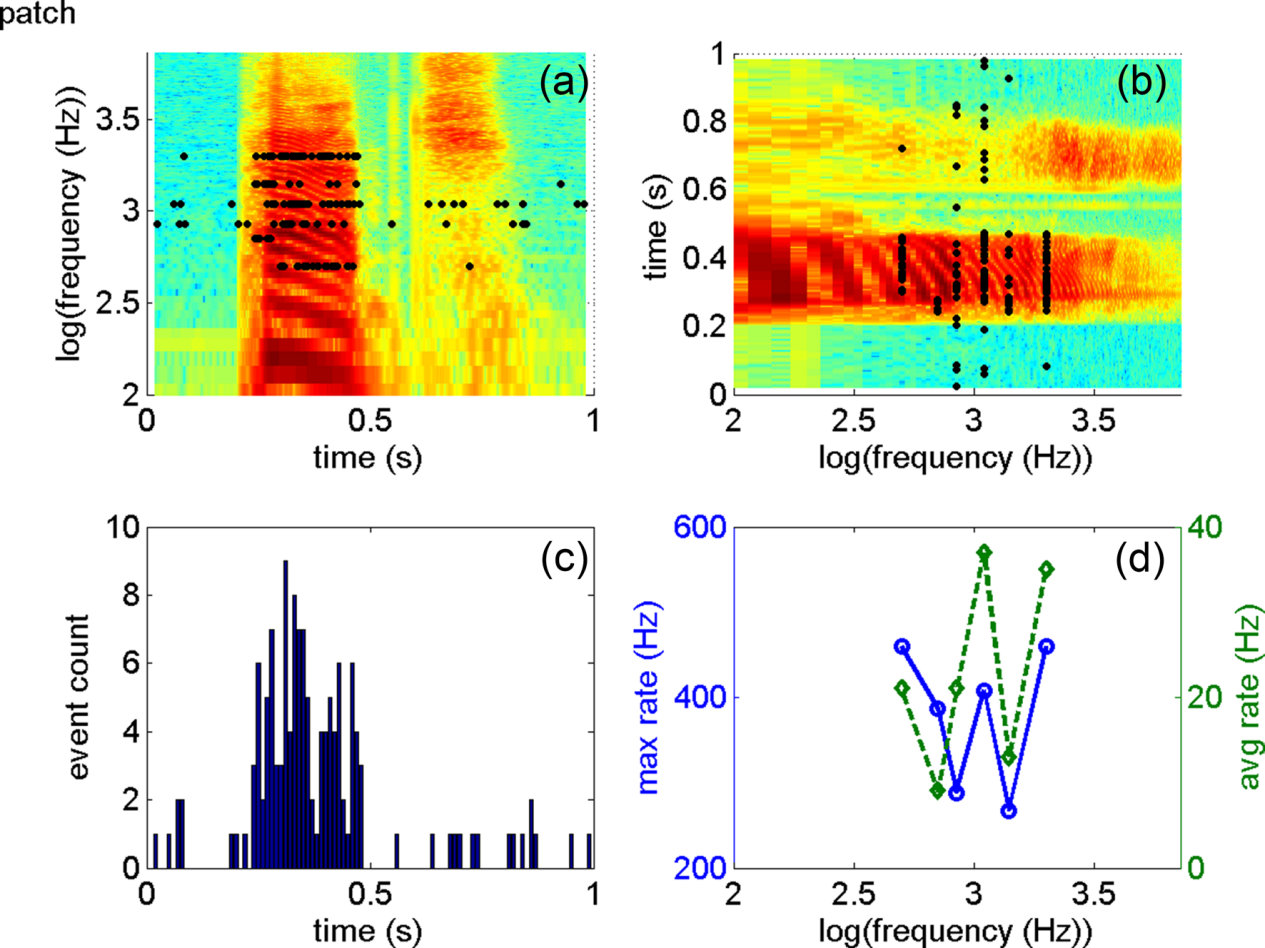
(**a**) Shows the spectrogram of the word “patch” overlaid by the black dots of the electrodogram (cochlear implant stimulation pattern). The electrodogram was constructed from the recordings in the guinea pig central nucleus of the inferior colliculus obtained while the word “patch” was played at an average sound level of about 60 dB SPL. (**c**) Shows the sum of the pulses in one bin (~ 22 µs). The more events are counted in a bin, the louder the stimulus will be. (**b**) Shows the spectrogram again with flipped axis. (**d**) Provides the corresponding average rate in one channel and the maximum rate as calculated from the shortest time between two pulses. The average rate is low. However, when stimulation occurs, it is between 300 and 500 Hz. The rate changes dynamically. Intensity pattern could be used as a cue in the processing of the encoded acoustic information.

At the beginning of the testing session, after the signal's loudness was adjusted to a comfortable hearing level (for details, see “Methods”), the first two subjects (S1 and S2) had to describe their hearing experience. S1 and S2 did not know that the sound signals were encoded words. They were able to discriminate length, rhythm, and loudness differences. Although the biphasic pulses' amplitude was constant so that the current amplitude did not encode loudness, the patients reported loudness differences within one word. An explanation is that the pulse rate across channels (Fig. 2a,c) and the pulse rate within one channel (Fig. 2b,d) sum and encode the sound level intensity. When told that the sound signals are words and the lexical content of the words they listened to during the training portion, both patients stated that what they heard sounded different.

During the following section of the testing session, both subjects knew that they were listening to words. They were not told the lexical content but had to communicate what they understood—each subject assigned lexical information to the information they received. While the patient’s answer was sometimes wrong, it remained the same across multiple trials. For example, when played the word “ditch,” one subject stated the word was “Willhelm.” When “ditch” was replayed without the subject’s knowing which word, they once again said that it sounded like “Willhelm.” Patients received, identified, and interpreted the same cues twice in the same way.

#### The lexical content of the transferred signal

**Figure 3:**
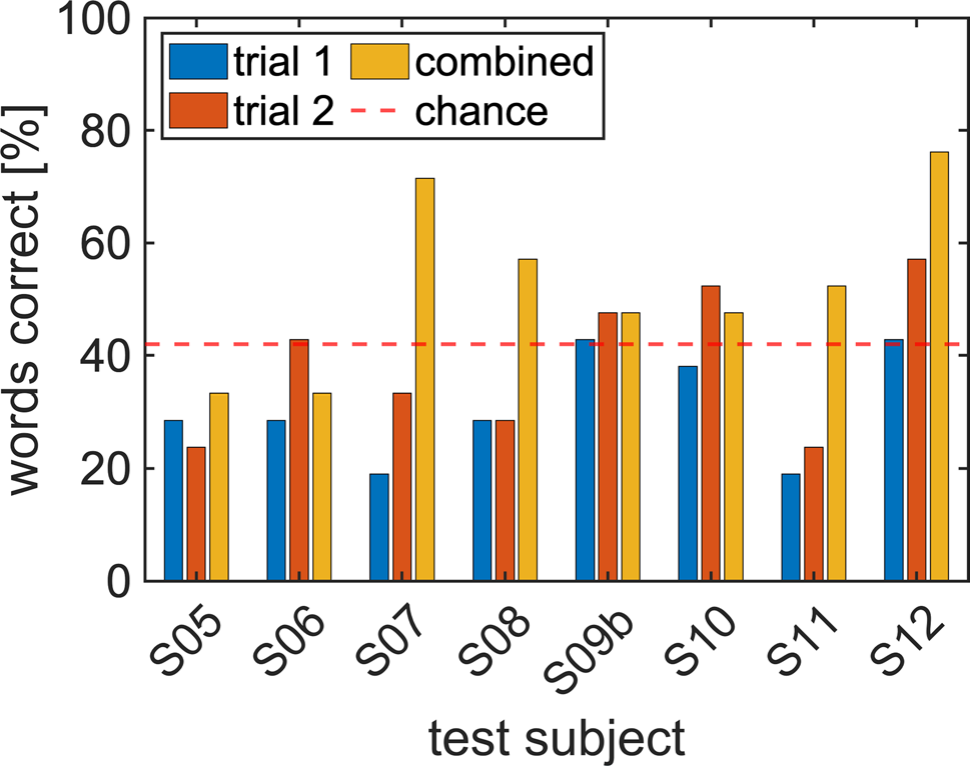
The results from each subject. Recognition scores often have higher than 50% recognition rates. Some test scores are near 50% correct, and for recognition, close to 80%. Chance was at 42.5%. Initially, only the BEDCS was available to us for testing. Subjects S1–S9 were tested with this system. The forced-choice comparisons were only completed in S5–S9. Table 1 (cumulative results) and STable 1 (individual results) show the subjects' performance on the test.

Subjects S5–S12 performed a four-word forced-choice test. The communication between the computer and the CI occurred with the Bionic Ear Data Collection System (BEDCS) in subjects S5 through S9 and with the HR-Stream in S6b, S7b, S9b, and S10 through S12. We used both systems for S6/S6b, S7/S7b, and S9/S9b. The test subjects listened to the signal transmitted via their CI and subsequently selected one out of four possible words. Each subject completed the word list twice, trial 1 and trial 2, in the same order. Subjects were not informed that and when the repeat started. The patients did not receive feedback on whether their answer was correct or wrong. Figure 3, Table 1, and STable 2 and STable 3 show each subjects’ performance on the test.

**Table 1:**
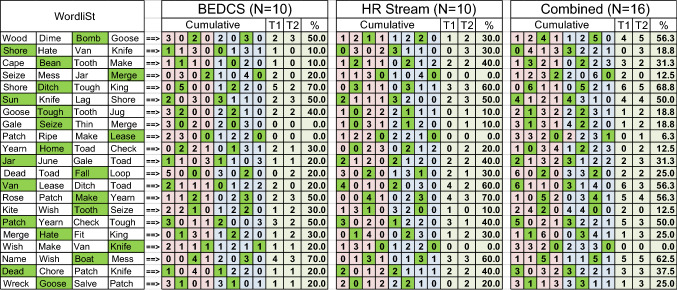
The left four columns show the 21 sets of four words shown to the test subject.

The word with the green background is the correct word in a set of four, matching with the information presented to the test subject via their cochlear implant. Words selected by the test subject are shown in the cumulative tables for the BEDCS, the HR-Stream, and the combined data. N indicates the number of times a word set is included in the analysis. For example, for the BEDCS system (N = 5), test subjects × 2 presentations of a word set, which equals 10. No feedback has been given to the patient on the correct answer. Therefore, the presentation of the same set twice was considered independent. For the nine columns assigned to each, “BEDCS”, “HR-Stream”, and “combined” the following applies: The four columns with the red backgrounds show the frequency a word was selected in trial 1 (T1), and the four columns with the blue background show how often a word was chosen in trial 2 (T2). The column with the header “T1” counts how often the correct word was selected in trial 1, the column with the heading “T2” shows the number of correct words selected in trial 2. The rows in the column “%” show the overall percentage correct for a given word.

**Figure 4:**
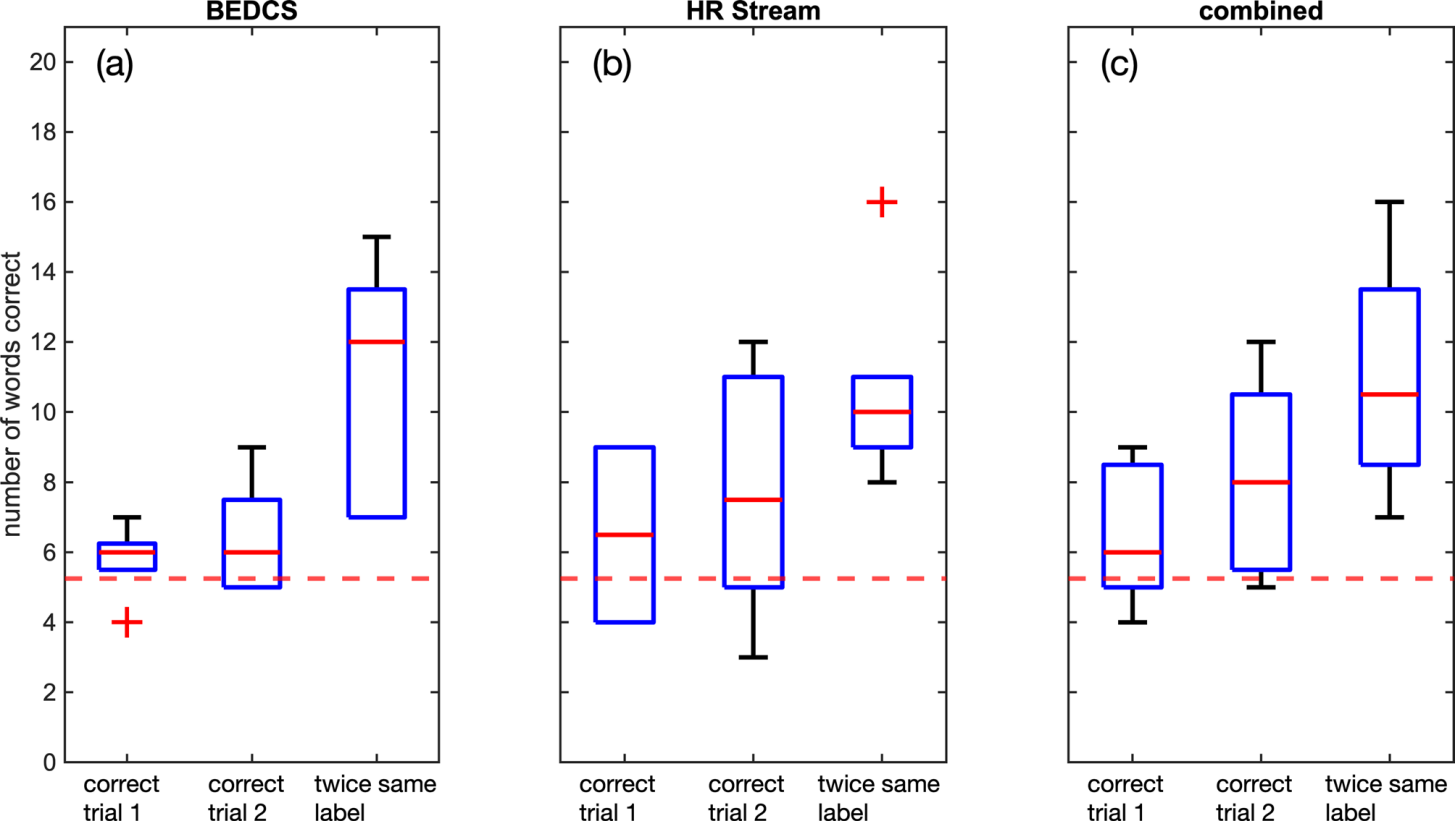
Subjects scored significantly better than choice. Trial 1 and trial 2 are the first and second run through the 21 forced-choice comparisons. The recognition test indicates the occurrence of selecting the same answer twice, right or wrong, on both trial 1 and trial 2 for each testing session. This may provide some insight into the ability to discern lexical information, even if it may be distorted. The test has four classes, and with the assumption of infinite sample size, the expected number of words correct is 21/4 (broken red line). Since the number of groups presented (samples) is not infinite, the statistical significance threshold was used.

Figure 4 shows the aggregate results, with Fig. 4a the results from test sessions done with the BEDCS; Fig. 4b the results from test sessions done with the HR-Stream. Figure 4c shows the results from the combined data (twice the same word). The ordinate provides the number of correct answers out of 21 questions. With the BEDCS, the correct answers were 28.2% in trial 1, 31.1% in trial 2. The performance was better with the HR-Stream, with 31.0% in trial 1, 36.5% in trial 2. In addition to scoring the correct answers, we also counted the selection of twice the same wrong word. This evaluation's rationale was that the subject must have recognized the selected word from their spectral and intensity cues but have given the wrong lexical content. With the BEDCS, twice selecting the same wrong word was 37.9%, and 30.2% with the HR-Stream. For the test subjects selecting a word twice (correct or false), the scores were 54.4% with the BEDCS and 50.8% with the HR-Stream. Feedback was not shared throughout the testing session, preventing the subjects from learning or making adjustments during the testing session.

**Figure 5:**
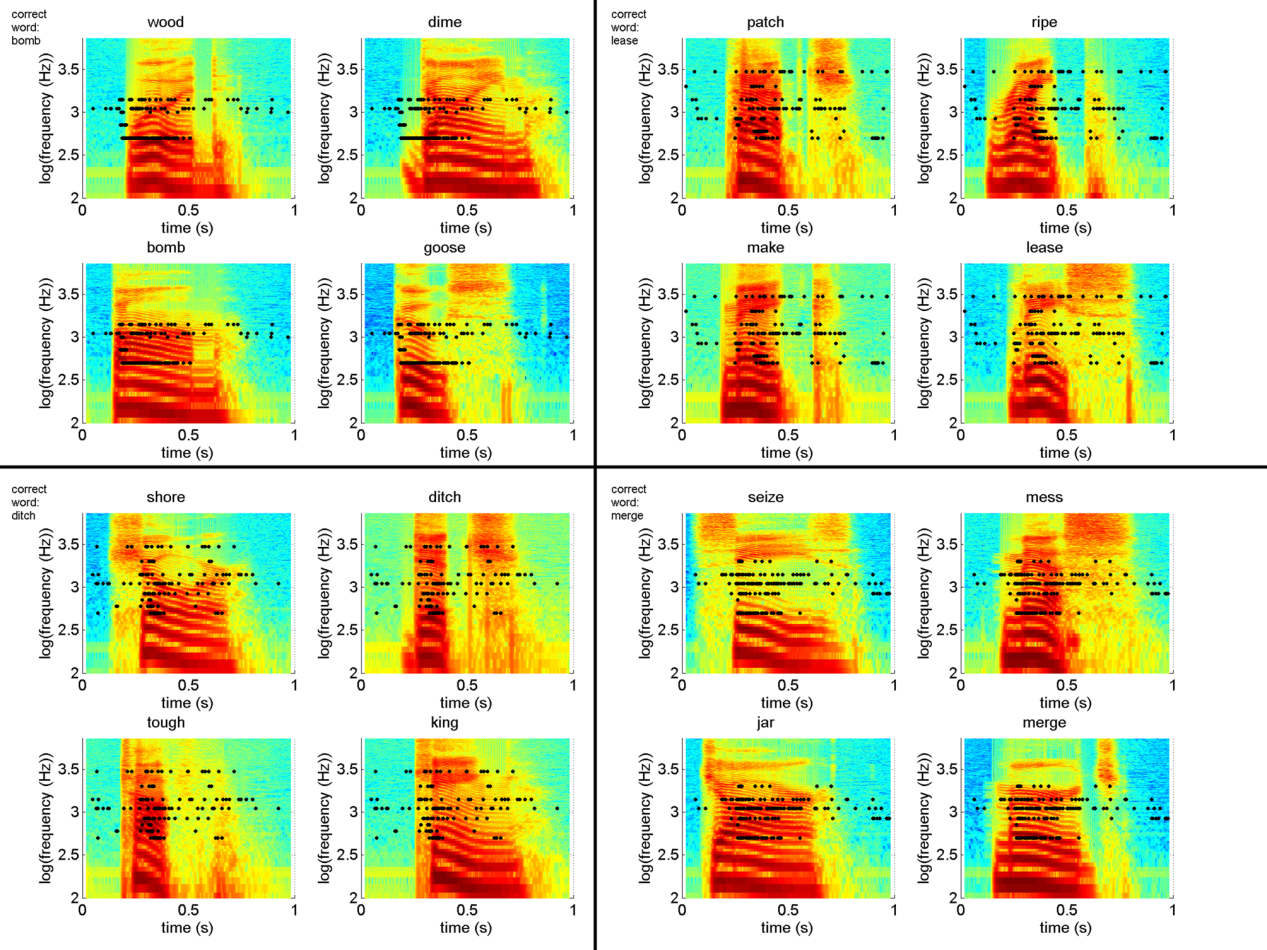
The 16 panels show the spectrogram of four sets of four words. The correct word is given at the left upper corner of each 2 × 2 set of words. On average, the words on the left (bomb and ditch) were identified correctly in over 50% of the cases, the two words on the right (lease and merge) were identified in less than 10% correctly. The raster plots for the correct word overlay each spectrogram in one set. Each black dot indicates the timing when a pulse is delivered. The spectrogram and electrodogram can be directly compared. From the figure, it is obvious that the frequencies and the timing are poorly matched. It is also clear from the electrodogram that the test person had cues from intensity patterns or length of the stimulation. The electrodograms also show that either an artificial code or a better-distributed pulse pattern is required to improve performance.

It should also be noted that the test subjects recognized some words better than others. The words selected correctly for more than 50% are bomb, ditch, sun, make, patch, boat with the BEDCS (STable 1) and ditch, sun, van, make, boat with the HR-Stream (STable 2). Test subjects performed especially poorly (≤ 10%) recognizing shore, bean, seize, and lease with the BEDCS (STable 1) and identified ≤ 10% of times the correct word merge, tough, seize, lease, tough, and knife with the HR-Stream (STable 2). Figure 5 shows the spike patterns recorded for the correct word together with the spectrograms of the set of four words presented during the forced-choice test. It is not apparent why one word should be favored over another. It is not clear why a test subject should perform better for the words bomb or ditch when compared with the words lease or merge.

#### Performance above chance

As described in “Methods”, we corrected the threshold for chance for the small sample size using the MATLAB function binoinv(). For an N of 21 and a four-word forced-choice test, performance for each subject was above chance if the score was 42.5% or more. The performance differs among the test subjects (Fig. 3). None of the subjects tested with the BEDCS performed above chance during their first trial (trial 1) and only S6 during the second trial (trial 2). For the same word selected twice, S7 and S8 scored above chance. The scores were higher with the HR-Stream. S9b (retested S9) and S12 performed above chance during trial 1; S9b, S10, and S12 scored above chance during trial 2, and S9b through S12 for the same word selected twice.

We tested the mean accuracy for all test subjects during trial 1, trial 2, and with a two-tailed binomial test (myBinomTest(); MATLAB_R2020b) if the number of questions answered correctly is above chance for trial 1, trial 2, and twice the same word (correct or wrong). Table 2 shows the results. The numbers are the probabilities to accept the hypothesis that the selection of the words during the forced-choice test is chance. The word recognition tasks' performance was above chance for the combined data set if the probability shown was smaller than 0.05.

**Table 2:** The MATLAB function myBinomTest() was used to test whether performance is above chance.

	myBinomTest()		
	Trial 1	Trial 2	Twice the same word
pBEDCS (p = 0.25)	0.5729	0.1236	0.1094
pHRS (p = 0.25)	0.2145	0.0039	0.0096
pComb (p = 0.25)	6.3E−09	6.6E−10	1.0E−21

Performance was not above chance for the test with BEDCS alone. With the HR-Stream, performance was above chance for the second trial and twice the same word. Performance was significantly above chance for the combined performance evaluation.

## Discussion

Overall, patients performed better than selecting the right answer by chance for test 2 and the recognition (twice the same word in trials 1 and 2, correct or wrong) test. On average, subjects performed noticeably better on the recognition test over testing for the accurate lexical content. These results indicate that a low-level speech perception occurs, though open-set speech perception has yet to be achieved. The acoustic signal, encoded by an animal's auditory system (guinea pig), can be deciphered by the human auditory system. This study's results have led to the development of a novel coding strategy to be implemented and used in CIs^7, 8^. Tests in human CI users using the novel coding strategy are currently ongoing.

CIs restored hearing in more than 550,000 implanted severely-to-profoundly deaf^9^. While some CI users' performance is exceptional, many users complain about poor performance in noisy listening environments, difficulties with tonal languages, and difficulties with music perception. What is missing? A normal-hearing listener's auditory system performs a spectral analysis of sound using an array of overlapping auditory filters. The output of each filter is like a bandpass filtered version of the sound. It contains two forms of information: the relative slow variation in amplitude over time (E, envelop) and the rapid oscillations with the rate close to the filter's center frequency (TFS, temporal fine structure). E cues alone can lead to high intelligibility for speech in quiet. However, in noisy listening environments and music recognition, normal hearing subjects also take advantage of TFS. Although it has been demonstrated in normal hearing subjects that temporal fine structure (TFS) is important for speech recognition in noise^10–17^, to process tonal languages^18^ and for music perception^19, 20^, most of today’s cochlear implant (CI) coding strategies rely primarily on the envelope (E, for more details, see below). With the limited success of the few coding strategies that specifically claim to encode TFS, it is important to determine whether CI patients could potentially improve in performance with TFS included in the coding strategy.

This study's results help progress by underlining that the neural activity recorded in an animal auditory nerve or midbrain can serve as a surrogate for a similar approach in a cochlear coding strategy. Based on the results from this study, we propose encoding E and TFS by modulating a stochastic pulse pattern with the center frequency of the selected frequency band. To model the frequency place-map along the cochlea, at each electrode contact, stochastic pulse trains with a Poisson-like distribution of inter-pulse-intervals are frequency-modulated, corresponding frequencies obtained from the location of the electrode contact along the cochlea. The current amplitude of the pulses is constant and does not contribute to loudness growth, which codes the spatial and temporal rate changes, as demonstrated in Fig. 2. Overall rate changes code E, and temporal correlations code TFS for each channel. This code has been developed recently and tested in a pilot study with 17 implant users with implants from 2 major CI manufacturers. The research is ongoing, and the results will be published elsewhere.

A limitation in the experimental design is using the forced-choice test because it provides the words in the first place. The performance is not necessarily speech recognition but the ability to select the best fit. Several phonetic cues are available to the test subject, including loudness changes, rhythm, and word length. To identify possible cues, all patients were asked at the beginning of the session to describe what they hear without being told that the signals they heard were single words. They characterized the words by their loudness changes, their rhythm, their length, and sound quality. When told that the signals they listened to words, they assigned lexical content or ‘label’ to the words. The lexical content could be right or wrong. When presented with the same word later in the session, 6 out of 8 test subjects would offer the same label in more than 50%.

It is noteworthy that the test subjects recognize the loudness variations despite the amplitude of the biphasic charge-balanced pulses and their pulse lengths were constant. Only the pattern of pulse occurrence changed.

The results also show that speech recognition depends on the frequencies selected. Unfortunately, the neurons recorded in the guinea pig ICC only cover parts of the given words' required frequency information (Fig. 5). In many of the examples, crucial frequency information is missing to distinguish between two words clearly. The importance of the number of frequencies or frequency bands for the performance in normal hearing, hard of hearing, or even cochlear implant users has been stressed before^21–26^. The number of available frequency bands will affect performance during the test. Studies with normal-hearing human subjects addressed how many independent channels are required to perceive combinations of multiple pitches or a single pitch with interfering tones^23, 24, 27^. 32–64 channels without any spectral overlap and a filter slope of at least 72 dB/octave are required for extracting spectral pitch. In a different set of experiments by Stafford and coworkers^27^ with normal hearing subjects presented with vocoded speech, the number of channels was fixed. The filter slopes were 2 dB/mm (~ 9.2 dB/octave), 10 dB/mm (~ 46 dB/octave), and 17 dB/mm (~ 72.8 dB/octave). Performance increased drastically when the filter slope was changed from 9.2 to 46 dB/octave. This is in contrast to the ability of the auditory system of a normal hearing subject with about 3500 hair cells (resulting in 50–100 independent channels^25^) and filter slopes of about 135 dB/octave (frequency range below the frequency of interest) and about 390 dB/octave (frequency range above the frequency of interest).

Test subjects in this study were cochlear implant users with a damaged auditory system and subsequent neural degeneration. Only a limited number of frequency bands could be used. Cochlear implant patients' speech performance, such as consonant recognition, is a function of the available number of channels^28^. In some patients, performance improved as the number of channels was increased up to 6. In this study, further increase in the number of channels did not improve performance^28^. Other studies demonstrated that CI users' sentence recognition increased from one to ten channels, but there was no difference in performance if more channels were used^21, 22, 26^. Overlapping electric current fields might have limited the number of channels during stimulation at neighboring electrodes, caused by factors such as electrode configuration, mode of stimulation (monopolar, bipolar), placement of the electrode array (insertion depth, distance between electrode and spiral ganglion neurons), or degeneration of the spiral ganglion along the cochlea. It is understandable that with a very rudimentary frequency representation from an animal speech performance is limited too—the low number of channels used while testing with the BEDCS device likely hinders speech recognition. Increasing the number of channels through the HR-Stream helped some patients boost their scores but resulted in no significant performance difference. This result does fit expectations since only 2–4 channels are needed for speech recognition, and more than four channels were always used^25^.

Another potential factor limiting patient understanding is the frequency map presented to the patients. Previously published work showed that the frequency-to-place mapping manipulations in the cochlea decrease speech recognition^29–33^. In the present study, the map used for patients S1 to S9 differed. The map presented to subject S9 during their second round of testing better matched typical Advanced Bionics provided frequency map of the CI the patients used with their implant. The map typically used in most implants may still be non-ideal and cause some degree of frequency distortion. From the plots where the pulse pattern is superimposed on the word's frequency presentation (spectrogram), it is clear that often information is missing, which could be an animal-human difference or the influence of anesthesia (Fig. 4).

Subjects S1 and S2, who were not tested using the forced-choice test, but simply asked to describe what they heard, had some training (< 2 h). All patients that completed the forced-choice test, though, had no degree of training. They were never told the correct word they were listening to and only heard the word presented 1–3 times on each forced-choice comparison. Adapting to understand a vastly different neural code, such as the one presented, may require significant training to boost the top-down mechanism of speech understanding. A more recent study showed that CI users identified CNC words correctly in a quiet listening environment in 47.2% at three months after the CI activation and 57.5% at one year^34^. Performance improved even after multiple years of use with their processing system. These results were achieved, allowing ample top-down mechanism^35–46^. Additionally, in this study, patients were given example words before testing to allow some training^47^. Since subjects were unaccustomed to this code, allowing for a training period could markedly improve speech perception.

## Conclusions

For the animal neural system to function as the basis for a sound processing system in cochlear implants, important speech cues must be accounted for. Currently used coding strategies mainly focus on place theory over volley theory, possibly ignoring the important dependence of speech on the neurons’ phase-locked responses. Processing speech via a functioning cochlea avoids this issue by naturally encoding speech and phase. It can be seen by analyzing the neural data's spectral representation by comparing the spectrogram to the corresponding raster plot. Mapping the recordings from a particular neuron to the tonotopic placement of that neurons’ CF on the cochlea acts as a spectral and waveform information extractor of sorts.

To encode loudness, typically done through amplitude modulation, the stimulation current remains constant for our experiments while the pulse rate increases. The patients reported loudness fluctuations while stimulated at continuous current. This may lead to an increase in the dynamic range resolution, though further studies are underway to verify this. Notably, while pulse rates vary, the maximum rate is still far below that used in current implants. This low rate stimulus can increase selectivity by limiting the current spread and increasing the neuron's spontaneous activity by stimulating at the threshold. Increased selectivity could lead to more genuinely independent channels, greatly benefiting speech intelligibility. Simultaneously, low rate stimulation could lead to longer battery life and compatibility with optics-based stimulation.

This study is encouraging. Over 50% recognition was achieved for multiple tests, and up to 70% achieved on the recognition test. This success was achieved even though the patient was only presented with a crude representation of the neural code: guinea pig neural responses recorded in the ICC that was successfully translated to a human auditory nerve. This transfer from a high processing center back to a lower one indicates that temporal fine structure (TFS, frequencies > 500 Hz) and temporal envelop (TE, frequencies ≤ 50 Hz) cues are transferable between the ICC and the auditory nerve and can help provide further insight into how auditory information is encoded. Additionally, improvements in the recordings' preparation and the recordings may significantly improve speech perception and allow the patient's training. This strategy could push cochlear implant technology to better speech in noise perception and music appreciation due to a more complex sound encoding. By allowing a perfectly functioning auditory system to analyze sounds, improvements can be made to speech processing either through using the system directly, as done in this study, or by analyzing the animal code to find new insights on how speech is encoded.

## Methods

### Ethics declaration

All animal procedures followed the NIH Guide for Care and Use of Laboratory Animals and received approval from the Institutional Animal Care and Use Committee at Northwestern University. The study was carried out in compliance with the ARRIVE guidelines. All experimental procedures with human subjects followed the institutional research committee's ethical standards and the 1964 Helsinki Declaration and its later amendments. The Institutional Review Boards at Northwestern University Feinberg School of Medicine and New York University School of Medicine reviewed and approved all procedures. The subjects gave informed consent before participating in this study.

### General description of the approach

The study's objective was to test whether neural responses to speech, recorded from the brainstem [central nucleus of the inferior colliculus (ICC)] in guinea pigs, can be deciphered by the human brain. To code speech composed of a complex pattern of different frequencies, neural activity from auditory neurons with a wide range of best frequencies is necessary. In this study, multi-channel recording electrodes were inserted into the ICC, recording many frequency bands simultaneously during speech presentations via a speaker. Each train of action potentials recorded at one of the electrode contacts in the ICC was then converted into a sequence of biphasic and charge-balanced electrical pulses with the same temporal pattern as the train of action potentials. The train of electrical pulses was presented to a cochlear implant user's brain via her/his cochlear implant. The amplitude of the electrical pulses is fixed in amplitude such that a comfort level of loudness was achieved. Single words encoded by the guinea pig’s auditory system were played to the test subjects, and they had to identify the word out of four (forced-choice). During the testing sessions, no feedback was given to the patient on whether they selected the correct word to reduce learning. The four words in a group were chosen such that additional information beyond the frequency information was minimized. In other words, loudness patterns, length, etc., were similar for the four words. Two systems were used to interface with the cochlear implants, the BEDCS and the HR-Stream. The second system allowed more channels and a better frequency representation of the speech signal.

### Animal data collection and preparation

#### Animals

Animal procedures are the same as we have used in the past and have been published previously^48–52^. We collected no new animal data; the animal data originate from a study on distorting temporal fine structure by phase-shifting, published in 2017 in Scientific Reports^52^. The recordings in four guinea pigs of either sex were suitable for this study. Animals 1, 3, and 4 were about seven months old, weighing 1045 g and 1090 g, and 860 g, and animal 3 was five weeks old and weighed 558 g. The following section describes the animal data collection briefly.

#### Animal anesthesia

A mixture of Ketamine (44–80 mg/kg) and Xylazine (5–10 mg/kg) was injected intraperitoneally to induce anesthesia. While under deep anesthesia, body temperature was maintained at 38 °C by placing the animal on a heated blanket. A tracheotomy was made, and a plastic tube (1.9 mm outer diameter, 1.1 mm inner diameter, Zeus Inc., Orangeburg, SC) was secured into the trachea. The tube was connected to an anesthesia system (Hallowell EMC, Pittsfield, MA), including a vaporizer (VetEquip, Pleasanton, CA) to maintain anesthesia with isoflurane (1–3%). During the experiments, the depth of anesthesia was assessed by a paw withdrawal reflex, and the isoflurane concentration was adjusted accordingly. Body temperature, breathing rate, and heart rate were monitored continuously and were logged every 15 min.

#### Placing the multi-channel ICC recording electrode

To access the ICC, the right temporalis muscle was reflected. An approximate 5 × 5-mm opening was made in the right parietal bone, just dorsal to the parietal/temporal suture and rostral to the tentorium. A small incision was made in the dura mater. A silicon-substrate, thin-film multichannel penetrating electrode array (A1 × 16-5 mm-100–177, NeuroNexus Technologies, Ann Arbor, MI) was advanced with a 3D-micromanipulator (Stoelting, Kiel, WI) through the occipital cortex into the ICC. The trajectory was dorsolateral to ventromedial at approximately 45º off the parasagittal plane in the coronal plane. The electrode array passed through the central nucleus of the ICC approximately orthogonal to its iso-frequency laminae^48, 53, 54^. After the initial placement of the electrode's distal tip into the ICC, the electrode was advanced while a pure tone stimulus was presented to the left ear. The final placement of the electrode was achieved when neural responses from the array's distal contact could be stimulated with a pure tone stimulus between 16 and 25 kHz. In some instances, the electrode was advanced several times into the ICC before the desired placement was achieved. After placing the electrode array, the exposed skull and dura mater were covered and protected from dehydration with gauze sponges (Dukal Corporation, Hauppauge, NY) soaked with Ringer’s lactated solution.

#### Pure tone and speech stimuli

Voltage commands for acoustic stimuli, generated using a personal computer (PC), equipped with an I/O board (KPCI 3110, Keithley, Cleveland, OH), drove a Beyer DT 770Pro headphone speaker (Beyerdynamic, Farmingdale, NY). The speaker's speculum was inserted with a short, 3 mm-diameter plastic tube into the opening to the cartilaginous ear canal. Acoustic stimuli were tone pips (12 or 20 ms duration, including a 1 ms rise/fall) with different carrier frequencies, presented at a rate of 4 Hz. We measured the speaker’s sound level at the speculum opening with an 1/8-inch microphone (Bruel & Kjaer North America Inc., Norcross, GA). In addition to pure-tone bursts, stimuli were single words from the Hearing in Noise Test (HINT) played via the Beyer DT 770Pro headphone speaker to the anesthetized guinea pigs.

#### Data acquisition and electrical pulse trains

We recorded the neural activity with a multi-channel electrode array and a Plexon data acquisition system (16-channel, Model MAP 2007-001, Plexon Inc, Dallas, TX) as described before^48^. Neural activity was recorded at a 40 kHz sampling rate at each channel, with a 16-bit analog/digital (A/D) input conversion. The recorded signal was bandpass filtered, 0.1–8 kHz. Times at which spikes occurred were determined online with Plexon’s data acquisition software and stored for all 16 active electrodes. Following filtering to remove the field potential response, a user-defined threshold determined the neural activity considered for further analysis. Neural activity was recorded at each site along the electrode array during the presentation of pure tone stimuli at different carrier frequencies and sound levels. Responses to pure tone stimuli were used to construct a tuning curve to determine the best frequency of each neuron recorded. The best frequency of a recording site was the pure tone stimulus frequency, which needed the lowest sound level for a response at this site. Words from the HINT test were played at about 60 dB average sound pressure level (SPL = sound level re 20 µPa). Recordings from 100 distinct neuronal units were used with frequencies from 500 Hz to 16,000 Hz. Recordings of the neural activity were accepted if the action potential amplitude had a signal to noise ratio of more than 6 dB and no apparent artifacts from the electrical signal from the heartbeat and artifacts caused by breathing.

**Figure 6:**
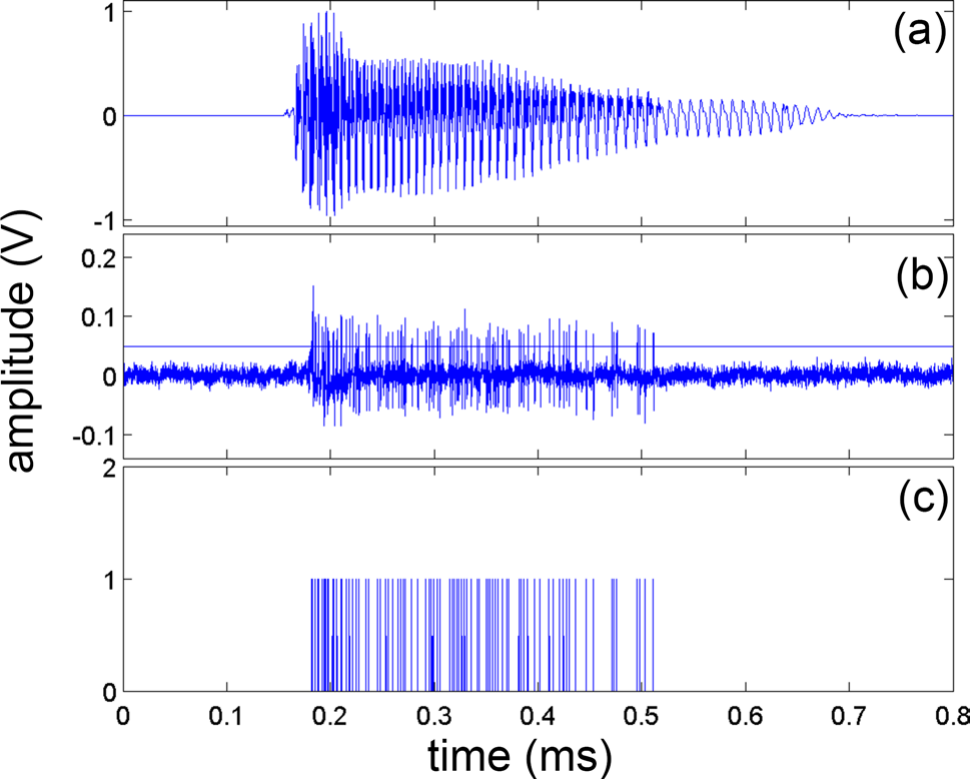
This figure shows the generation of a spike train from the neural recordings of the word “bomb.” (**a**) Shows the waveform of the word. (**b**) Shows a recording from a neural unit with a characteristic frequency of 500 Hz. The amplitude in (**b**) is 1000 times amplified. The threshold amplitude was at y = 0.05 (**b**), and each crossing of the threshold from low-to-high is an action potential or spike. (**c**) Shows the resulting spike train. At each peak in the spike train, a charge-balanced biphasic pulse was generated and presented through a cochlear implant. The best frequency of the spike sequence was matched as good as possible with the frequency place map established for the cochlear implant user.

The recorded audio files were then converted to a vector containing timing information for an action potential. The vector had zeros and ones, with ones encoding the time when the recording crossed a predefined threshold level from low level to high. An overview of the spike train generation can be seen in Fig. 6.

**Figure 7:**
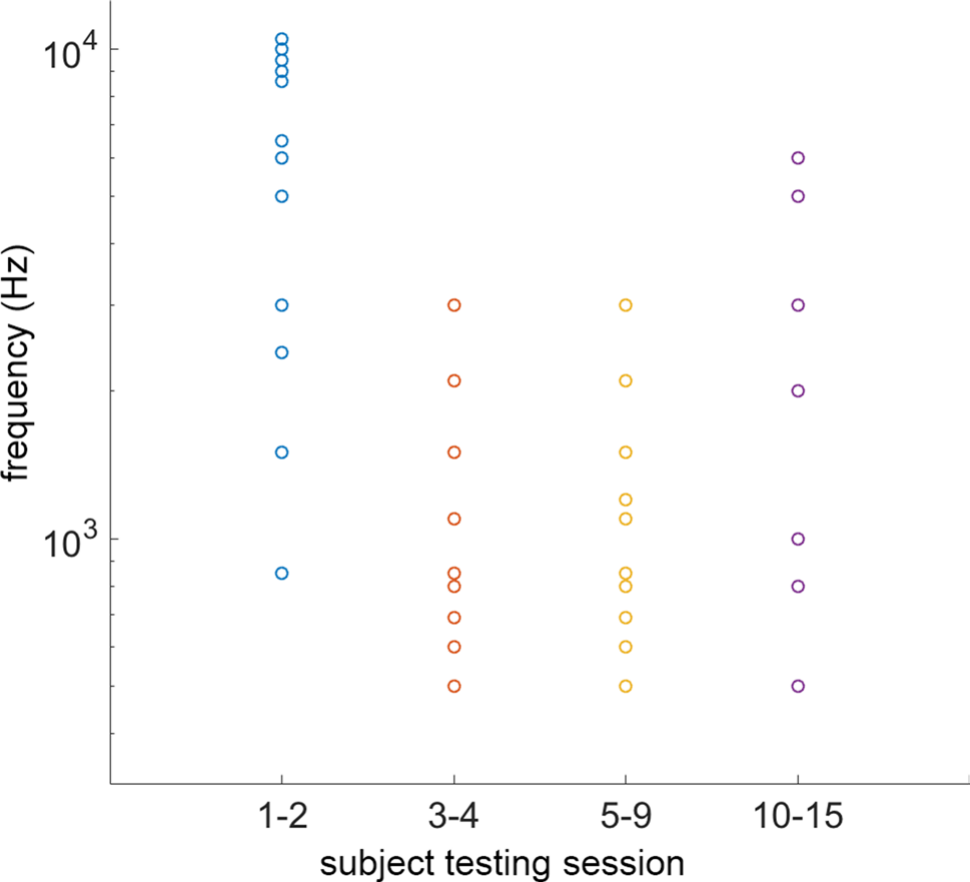
The figure shows the maximum number of channels used during the testing sessions. Each circle represents a channel used to deliver the processed neural recordings across. The frequency of that channel corresponds to the characteristic frequency (CF) of the originally recorded neural single unit. A channel is defined here as an electrode contact with the most basal contact corresponding to electrode 1 and the most apical contact corresponding to electrode 16. For testing sessions 1–4, the frequencies, from lowest to highest, were played to electrodes 1, 2, 3, 4, 6, 7, 8, 10, 11, 12, 13 and 14. For words that required less than 12 channels, the most apical (i.e., highest frequency) channels were removed. For testing sessions 5 to 9, the frequencies were played to electrodes 1, 2, 3, 4, 6, 7, 8, 10, 11 and 12. Once again, for words requiring fewer channels, the most apical channels were removed. For session 10, all words were played across only six channels played to electrodes 1, 6, 7, 11, 13, and 15. Finally, for the remaining testing sessions, 11–15, all words were played across 15 channels corresponding to electrodes 2 to 16. The final frequency map used for testing in sessions 10 to 15 was closest to the standard AB patient map used in our subjects’ current CI’s.

Each spike train corresponds to a neuron with a CF that was then used to determine the closest electrode to stimulate based on the tonotopic coding of the cochlea. The frequency distribution among the 16 available electrodes in the Advanced Bionics implant changed over the process of the experiment, as shown in Fig. 7.

The map is non-ideal both due to the inability to obtain ICC recordings at every desired frequency and due to limitations of the first device (BEDCS) used for subject testing (subjects 1 through 9). The device lacked the random-access memory (buffer) necessary to present across all 16 channels simultaneously. Thus between 6 and 12 channels were active depending on the word presented to the initial nine subjects. The maximum pulses per second were calculated and averaged across all files presented to patients. Similarly, the instantaneous rate, defined as the rate between two successive pulses, was also calculated for all files. The instantaneous rate was limited to below 1.4 kHz due to the spike trains' necessary down sampling to fit the RAM limitations of the device. After switching to a new and less limited device (HR-Stream), the instantaneous rate became 2.4 kHz. The cross-correlations of spike trains delivered to each channel were calculated and graphed. The pulse trains presented to adjacent channels were cross-correlated to assess their stochasticity.

### Subject testing

To assess the recordings' lexical content, adult cochlear implant users (n = 12) were played the trains of electrical pulses and asked to complete specific tasks. Patient demographics can be viewed in Table 3.

**Table 3:** Subject demographics for each testing session.

Testing session	Subject tested	Age (years)	Gender	Implant TYPE (R)	Implant type (L)	Processor type	Implant location	Ear tested	System tested	Forced choice
1	S1	74	Male	HR90K/HiFocus 1 J	HR90K/HiFocus 1 J	Nadia CI Q70	Bilateral	Right	BEDCS	NO
2	S2	64	Female	–	HR90K Advantage/HiFocus ms	Nadia CI Q70	Left	Left	BEDCS	No
3	S3	66	Male	HR90K/HiFocus 1 J	–	Harmony	Right	Right	BEDCS	Yes
4	S4	80	Female	HR90K Advantage/ HiFocus ms	–	Nadia CI Q70	Left	Left	BEDCS	Yes
5	S5	50	Female	None	HR90K/HiFocus 1 J	Nadia CI Q70	Left	Left	BEDCS	Yes
6	S6	47	Male	HR90K/HiFocus 1 J	None	Nadia CI Q70	Right	Right	BEDCS	Yes
7	S7	55	Female	HR90K/HiFocus 1 J	HR90K/HiFocus 1 J	Nadia CI Q70	Bilateral	Left	BEDCS	Yes
8	S8	63	Female	None	HR90K/HiFocus 1 J	Nadia CI Q70	Left	Left	BEDCS	Yes
9	S9	50	Male	HR90K/HiFocus 1 J	CII/HiFocus 1 J	Nadia CI Q70	Bilateral	Left	BEDCS	Yes
10	retest S9	–	–	–	–	–	–	–	BEDCS	Yes
11	S10	71	Male	*na*	*na*	*na*	*na*	Right	HRStream	Yes
12	S11	85	Male	*na*	*na*	*na*	*na*	*Right*	HRStream	Yes
13	S12	71	Female	HR90K/HiFocus 1 J	CII/HiFocus 1 J	Nadia CI Q70	Bilateral	Left	HRStream	Yes
14	retest S7	–	–	–	–	–	–	–	HRStream	Yes
15	retest S6	–	–	–	–	–	–	–	HRStream	Yes

Sessions 10, 14, and 15 were retests of previously tested individuals. The entry of “*na”* indicates that a patient did not have their implant information available to provide us with the relevant testing details.

Of the 12 patients, six were female, and six were male with a mean age of 65 ± 12 years. Testing of the first nine patients was conducted using the Bionic Ear Data Collection System (BEDCS). The three new patients and two returning patients were tested with the HR-Stream, both devices on loan from Advanced Bionics. The loudness of the stimuli was adjusted globally, starting from 0 µA of current and increased until a comfortable listening level was reached. Comfort was defined using a standard audiology scale that ranges from 0 to 10, where 0 indicates no auditory perception, 10 extreme discomfort, and 5 indicates a comfortable listening level.

During the first two testing sessions, subjects S1 and S2 were played 21 different words with between 6 to 12 channels used to present the information, varying based on the word presented. The frequency maps used for testing sessions 1 and 2 can be seen in Fig. 2. The first part of testing consisted of a training session where the subject was informed of the word they were being played. After the training session, subjects were played words without being told which word they were hearing. They were asked to describe what they heard and if they could identify the word.

The testing protocol changed after the session. During sessions 2 and 3, the test subjects S3 and S4 were asked once again to describe what they heard and if they could identify the word with no context. After that, they were asked to complete a forced-choice comparison amongst four different words. One of the four words corresponded to the word being presented to the patients. The same set of 21 words was presented using the same frequency map as in testing sessions 1 and 2. At this point, subjects were no longer provided with training before presenting each word. Subjects were not informed of the correct answer after making the forced-choice comparison. After completing the first trial (T1) of 21 force choice comparisons, S3 and S4 were asked to retest a few words of interest.

For the remaining testing sessions 5–15, subjects were given a standardized list of 21 forced-choice comparisons. Once again, no training was provided, and the subjects were not given the correct answer. Subjects were also asked to take a retest (trial 2) of the 21 forced-choice comparisons. Subjects 5 and 6 completed only 20 out of the 21 forced comparisons due to a technical issue during testing. The frequency map used to present these words to the subjects was altered as more data was collected; each map can be seen in Fig. 7. For sessions 5 to 9, between 6 and 10 channels were used to present information, varying based on the word presented. For session 10, the protocol remained the same though the frequency map was shifted, as seen in Fig. 7, and only 6 channels were used to present each word to the subject. Additionally, the protocol remained the same for testing sessions 11 to 15. With the HR-Stream system in use for these sessions, subjects were presented with information across 15 channels for every word, and the altered frequency map can be seen in Fig. 7.

### Data analysis

The threshold for chance was calculated to 25% by dividing the 100% by the number of classes (c), four for this study. However, this is only valid for large sample sizes^55, 56^. For smaller sample sizes with an N of 21, such as in our test, the threshold for chance was corrected using the MATLAB function binoinv()^55^; threshold = binoinv(1-alpha,N,1/c)*100/N, where alpha = 0.05, c = 4, and N = 21. The resulting threshold was 42.5%, indicating that the test subjects using the BEDCS performed less than chance.

For each testing session, trials 1 and 2 were scored to provide the number correct for each subject. The trials were also scored for recognition (same label twice); the amount “recognized” is defined as an answer that was the same across both test 1 and test 2 despite if it was right or wrong. The number of questions answered correctly was tested using a two-tailed binomial test (myBinomTest(); MATLAB). The results were tested at a significance level of α = 0.05.

## Supplementary Information

Supplementary Tables.

## Data Availability

The data generated or analyzed during this study are included in this published article (and its Supplementary Information files). The datasets generated during and analyzed during the current study are also available from the corresponding author on reasonable request.
