# Supplementary material for: Listening to speech with a guinea pig-to-human brain-to-brain interface
Source: Sci Rep. 2021 Jun 10;11:12231. doi: 10.1038/s41598-021-90823-1 (PMC8192924; doi:10.1038/s41598-021-90823-1)
Supplement: Supplementary file 1 — Supplementary Tables. [file 41598_2021_90823_MOESM1_ESM.docx]

**Listening to speech with a guinea pig-to-human brain-to-brain interface.**

**-- Supplemental Materials --**

Claus-Peter Richter^1-4^, Petrina La Faire^1,2^, Xiaodong Tan^1^, Pamela Fiebig^1^, David M. Landsberger^5^, Alan Micco^1,4^

^1^ Department of Otolaryngology, Northwestern University, 320 E. Superior Street, Searle 12-561, Chicago, IL 60611, USA

^2^ Department of Biomedical Engineering, Northwestern University, 2145 Sheridan Road, Tech E310, Evanston, IL 60208, USA

^3^ Department of Otolaryngology, New York University, School of Medicine, 550 First Avenue, New York, NY 10016, USA.

^4^ Center, Department of Communication Sciences and Disorders, Northwestern University, Evanston, IL 60208, USA.

^5^ The Hugh Knowles Center for Clinical and Basic Science in Hearing and Its Disorders at Northwestern University, Evanston, IL 60208, USA.

**Short running title:** Neural coding using an animal model.

**Address**:

Claus-Peter Richter, M.D., Ph.D.; Northwestern University Feinberg School of Medicine; Department of Otolaryngology; Searle Building 12-470; 303 E. Chicago Avenue; Chicago, IL 60611-3008; U.S.A.; Phone: (312) 503 1603; FAX: (312) 503 1616; e-mail: [cri529@northwestern.edu](mailto:cri529@northwestern.edu).

# **Acknowledgements**

This project has been funded in part by a Northwestern University McCormick Undergraduate Research Grant, and in parts by the NIH/NIDCD, grant R01-DC011855. We thank Dr. Natalia Stupak for help in collecting some of the pilot data. Additional support was provided by Advanced Bionics for their loan of both auditory research systems used for this study and subsequent technical support.

|  |
| --- |
| **STable 1.** Shown are the outcomes obtained with the Bionic Ear Data Collection System (BEDCS). The design of the system limited the number of frequency bands used simultaneously. The light red background (four left columns for each patient is the first presentation of the 21 words. The light blue background is the second presentation of the wordlist. “1” indicate the word selected from the list in STable1. “0” indicates that the word was not selected. If the word was correct it received a green background, and a yellow one if the selection was wrong. |

|  |
| --- |
| **STable 2.** Shown are the outcomes obtained with the HRStream cochlear implant interface. This device allowed processing more channels in parallel than the BEDCS. The light red background (four left columns for each patient is the first presentation of the 21 words. The light blue background is the second presentation of the wordlist. “1” indicate the word selected from the list in STable1. “0” indicates that the word was not selected. If the word was correct it received a green background, and a yellow one if the selection was wrong. |

|  |
| --- |
| **STable 3.** Shown are the outcomes obtained with the BEDCS and with the HRStream cochlear implant interface. Three patients were tested with both the BEDCS and the HRStream system. For the patients only the data obtained with the HRStream were included. This device allowed processing more channels in parallel than the BEDCS. The light red background (four left columns for each patient is the first presentation of the 21 words. The light blue background is the second presentation of the wordlist. “1” indicate the word selected from the list in STable1. “0” indicates that the word was not selected. If the word was correct it received a green background, and a yellow one if the selection was wrong. |
